# Indian Jujuba Seed Powder as an Eco-Friendly and a Low-Cost Biosorbent for Removal of Acid Blue 25 from Aqueous Solution

**DOI:** 10.1155/2014/184058

**Published:** 2014-10-14

**Authors:** L. Sivarama Krishna, A. Sreenath Reddy, W. Y. Wan Zuhairi, M. R. Taha, A. Varada Reddy

**Affiliations:** ^1^Geology Programme, School for Environmental Sciences and Natural Resources, Faculty of Science and Technology, Universiti Kebangsaan Malaysia, 43600 Bangi, Selangor, Malaysia; ^2^Analytical Division, Department of Chemistry, Sri Venkateswara University, Tirupati, Andhra Pradesh 517 502, India; ^3^Department of Civil and Structural Engineering, Universiti Kebangsaan Malaysia, 43600 Bangi, Selangor, Malaysia; ^4^Institute for Environment and Development (LESTARI), Universiti Kebangsaan Malaysia, 43600 Bangi, Selangor, Malaysia

## Abstract

Indian jujuba seed powder (IJSP) has been investigated as a low-cost and an eco-friendly biosorbent, prepared for the removal of Acid Blue 25 (AB25) from aqueous solution. The prepared biomaterial was characterized by using FTIR and scanning electron microscopic studies. The effect of operation variables, such as IJSP dosage, contact time, concentration, pH, and temperature on the removal of AB25 was investigated, using batch biosorption technique. Removal efficiency increased with increase of IJSP dosage but decreased with increase of temperature. The equilibrium data were analyzed by the Langmuir and the Freundlich isotherm models. The data fitted well with the Langmuir model with a maximum biosorption capacity of 54.95 mg g^−1^. The pseudo-second-order kinetics was the best for the biosorption of AB25 by IJSP, with good correlation. Thermodynamic parameters such as standard free energy change (Δ*G* ^0^), standard enthalpy changes (Δ*H* ^0^), and standard entropy changes (Δ*S* ^0^) were analyzed. The removal of AB25 from aqueous solution by IJSP was a spontaneous and exothermic adsorption process. The results suggest that IJSP is a potential low-cost and an eco-friendly biosorbent for the AB25 removal from synthetic AB25 wastewater.

## 1. Introduction

Many industries including textiles, printing, and paper and dye houses make use of dyes extensively. A practical problem, involving use of dyes, is the discharge of wastewater, containing dye, into the natural rivers, streams, and channels from these industries like textile, paper, leather, and distillery [1]. Wastewater resulting from dyeing processes is highly coloured and hot and is alkaline in nature, besides containing high quantity of dissolved solids [2]. Dyes have an effect on the aquatic life and consequently on the food web, even if present in low concentrations. The indiscriminate discharge of the wastewater causes damage to the environment, as the dyes contained in it are having low biodegradability and they are carcinogenic in nature and are toxic to the aquatic as well as human life [3].

One of the effects of discharge of colored wastewater is on its interference with transmission of sunlight into streams, thereby reducing the photosynthetic activity of aquatic life, besides affecting the aesthetic nature. Most dyes are very stable to activities like photo degradation and biodegradation or the effects of oxidizing agents [4, 5].

The types of dyes used in the industries include cationic (basic dyes), anionic (direct, acid, and reactive dyes), and nonionic dyes. Usually, even a very small amount of dye present in water is highly visible, even at very low concentrations.

Many physical and chemical methods such as sonochemical degradation, photochemical degradation, electrochemical removal, electrochemical degradation, coagulation and flocculation, membrane separation, activated carbon adsorption, biodegradation, fenton biological treatment scheme, photo-fenton processes, oxidation, or ozonization process are used for removal of dyes from wastewater. More or less these methods suffer some drawbacks [6]. The use of activated carbons, due to their high adsorption capacity, as adsorbents for dyes is common and they are very effective for this purpose. Practically, however, the use of activated carbons for adsorption has been limited, because of the associated problems including the disposal. The regeneration process is also quite expensive.

Activated carbons are very costly, as proved by many research studies, activated carbons have been used widely for removal of dyes from wastewater. Low-cost agricultural and forest materials and products have been used by researchers as adsorbents. Some of these include cattail root [4], spent brewery grains [5], bagasse pith [8], jute stick powder [16], hazelnut shells [7, 17], pecan nut shell [18], sugarcane bagasse [19], guava (*Psidium guajava*) leaf powder [20], jujuba seeds [21], jackfruit peel [22], orange peel [23], soy meal hull [24], peanut husk [25], pine cone powder [26], aleppo pine [27], gulmohar [28], sugar beet pulp [29], pine cone biomass of* Pinus radiata* [30], pine cone [31], cashew nut [32], oak sawdust [33],* Pinus sylvestris* [34],* Capsicum annuum* [35], princess tree leaf [36],* Potamogeton crispus* [37], and peanut husk [38].

Recent studies have focused on the use of IJSP, with much success, for removal of Congo red, a direct dye, from the aqueous solutions [39]. In the present work, an effort has been made of verifying the capability of IJSP as a means of removal of acidic dye, Acid Blue 25 (AB25), from the wastewater solutions, which are synthetic. This is necessitated by the fact that agricultural waste and by-products find it difficult to remove acidic dyes from the wastewater solutions.

## 2. Materials and Methods

### 2.1. Adsorbate

**Figure 1:**
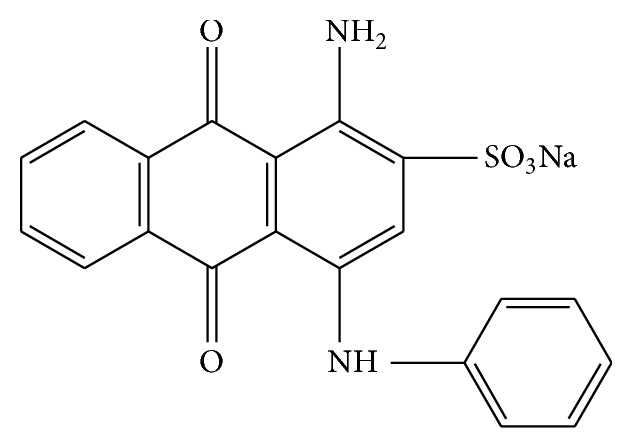
Chemical structure of Acid Blue 25.

The AB25 used in this study was purchased from Sigma-Aldrich, Hyderabad, India. Its C.I. number is 62055 and its chemical formula = C_20_H_13_N_2_NaO_5_S (FW = 416.38 g) and *λ*_max⁡_ = 610 nm. The chemical structure for AB25 was shown in Figure 1. The chemical was used without any further purification. Stock solution of 1000 mg L^−1^ was prepared by dissolving accurate quantity of the AB25 in double distilled water. The experimental solution was obtained by diluting the stock solution to the designed initial AB25 concentration.

### 2.2. Preparation of Biosorbent (IJSP)

The method of preparation of biosorbent is available elsewhere [39]. It is briefly explained here also. For the collection of Indian jujuba fruits, the location selected was Somayajulapally village which is very near to Nandyal town of Kurnool District in AP. The village is located in close proximity to the Nallamala forest, a popular forest range of AP. The flesh of these fruits was removed and a through washing with tap water followed. The washed seeds were exposed to sun and were dried. After that, the crushing of the seeds followed and the material was sieved with the help of sieves (mesh sizes varying from <53 *μ*m to <150 *μ*m). The prepared sample was stored in an airtight container, for use in the further studies. No other treatments, either physical or chemical, were adopted before carrying out the experiments on biosorption. The IJSP belongs to the Rhamnaceae family having the scientific name of* Ziziphus mauritiana*.

### 2.3. Biosorbent (IJSP) Characterization

#### 2.3.1. Point of Zero Charge (pH_PZC_)

The method of determination of point of zero charge of IJSP is described elsewhere [39].

#### 2.3.2. FTIR Analysis

Fourier transform infrared spectroscopy (FTIR) spectra of pristine IJSP biomass and the biomass loaded with AB25 were obtained by Thermo Nicolet, Nexus 670 Spectrometer, and resolution 4 cm^−1^. Pressed pellets were prepared by grinding the powder specimens with IR grade KBr in agate mortar.

#### 2.3.3. Scanning Electron Microscopy

Scanning electron microscopy of IJSP before and after adsorption is visualized by using Hitachi S-3000N scanning electron microscope (SEM).

### 2.4. Batch Kinetic Studies

Biosorption experiments were carried out by agitating 100 mg of biosorbent, IJSP with 25 mL of AB25 solutions of desired concentration, and pH in a 50 mL screw type Erlenmeyer flask at room temperature (35°C). A good contact is made between biosorbent and AB25 by agitating at 180 rpm in a Julabo shaking water bath. AB25 concentration is determined spectrophotometrically by monitoring the absorbance at 610 nm using Chemito UV-VIS Spectrophotometer, India. The wavelength of the maximum absorbance for AB25 is selected, and *λ*_max⁡_ value is 610 nm. The pH of AB25 solutions is determined using pH meter (Systronics pH system 361 Model, India). The samples were withdrawn from the shaker at predetermined time intervals and the AB25 solution was separated from the biosorbent by centrifugation at 10,000 rpm for 20 min. The absorbance of supernatant solution was measured, from which the amount of AB25 adsorbed was calculated.

The amount of biosorption at time *t*, *q*_*t*_ (mg g^−1^), was calculated using

(1) $$\begin{array}{c} q_{t}=\frac{\left[\left(C_{0}-C_{t}\right)V\right]}{W}, \end{array}$$

where *C*_0_ and *C*_*t*_ (mg/L) are the liquid phase concentrations of AB25 at initial and at any time *t*, respectively. *V* is the volume of the solution (*L*) and *W* is the mass of dry biosorbent used (*g*).

The AB25 removal percentage can be calculated as follows:

(2) $$\begin{array}{c} \text{Removal percentage}=\left[\frac{\left(C_{0}-C_{e}\right)}{C_{0}}\right]\times 100, \end{array}$$

where *C*_*e*_ is the equilibrium concentration in solution (mg L^−1^).

#### 2.4.1. Effect of Temperature

The effect of temperature on the amount of AB25 removal was studied at four different temperatures of 35, 45, 55, and 65°C. In this study, 25 mL of AB25 solution of 50 mg L^−1^ was taken into a screw type Erlenmeyer flask and agitated at 180 rpm, with 0.1 g of <53 *μ*m of IJSP at different temperatures. The AB25 solution pH was maintained in this study at 5.44. The samples were withdrawn from the shaker at predetermined time intervals and the AB25 solution is separated from the biosorbent by centrifugation at 10,000 rpm for 20 min. The absorbance of supernatant solution was measured. The amount of AB25 adsorbed was calculated by using (1).

### 2.5. Effect of IJSP Dose

The effect of IJSP dose on the amount of AB25 adsorbed was obtained by adding different amounts of IJSP (10, 30, 50, 80, 100, 150, 200, and 250 mg) into a number of 50 mL Stoppered glass Erlenmeyer flasks containing a definite volume (25 mL in each case) of fixed initial concentration (50 mg L^−1^) of solution without changing pH (5.44) at temperature, 35 ± 1°C. The flasks were placed in a thermostated Julabo shaking water bath and agitation was provided at 180 rpm for 180 minutes. After equilibrium, the biosorbent was separated from the adsorbent by centrifugation at 10,000 rpm for 20 min. The absorbance of supernatant solution was measured. The amount of AB25 adsorbed was calculated by using (1).

### 2.6. Desorption Studies

The biosorbent (100 mg) that was used for the adsorption of 50 mg L^−1^ of AB25 solution was separated from the AB25 solution by centrifugation. The AB25-loaded biosorbent was washed gently with water to remove any unadsorbed AB25. Several such samples were prepared for AB25. Then the spent biosorbent was agitated with 25 mL of distilled water, adjusted to different pH values for 180 minutes. The desorbed AB25 was estimated as before by using (1).

## 3. Theory of Biosorption Kinetics and Isotherms

### 3.1. Kinetic Models

The Lagergren pseudo-first-order model (3) and pseudo-second-order model (4) [39] have been widely used to predict sorption kinetics. The pseudo-first-order equation is generally applicable, over the initial stage of the biosorption processes, whereas the pseudo-second-order equation predicts the behavior over the whole range of biosorption. These two models were used in this study, to fit the experimental data:

(3) $$\begin{array}{c} \log\left(q_{e}-q_{t}\right)=\log q_{e}-\left(\frac{k_{1}}{2.303}\right)t, \end{array}$$

(4) $$\begin{array}{c} \frac{t}{q_{t}}=\left(\frac{1}{k_{2}q_{e}^{2}}\right)+\left(\frac{1}{q_{e}}\right)t, \end{array}$$

where *k*_1_ (min^−1^) is the rate constant of pseudo-first-order biosorption and *k*_2_ (g mg^−1^ min^−1^) is the rate constant of pseudo-second-order biosorption. *q*_*e*_ is the amount of AB25 adsorbed on biosorbent at equilibrium.

### 3.2. Intraparticle Diffusion Model

In order to investigate the mechanism of the AB25 biosorption, onto IJSP, intraparticle diffusion based mechanism was studied. The most commonly used technique for identifying the mechanism, involved in the biosorption process, is by fitting an intraparticle diffusion plot. It is an empirically found functional relationship, common to most of the biosorption processes, where uptake varies almost proportionally with *t* ^0.5^ rather than with the contact time *t*. According to the theory, proposed by Weber and Morris [22, 39],

(5) $$\begin{array}{c} q_{t}=k_{pi}t^{0.5}+C_{i}, \end{array}$$

where *k*_*pi*_ (mg g^−1^ min^−0.5^), the rate parameter of stage *i*, is obtained from the slope of the straight line of *q*_*t*_ versus *t* ^0.5^, whereas *C*_*i*_ is the intercept of the plot which gives an idea about the thickness of the boundary layer.

### 3.3. Thermodynamic Parameters

Thermodynamic parameters were calculated using the following equations:

(6) $$\begin{array}{c} \Delta G^{0}=-RT\ln K_{c}, \end{array}$$

where

(7) $$\begin{array}{c} K_{c}=\frac{C_{s}}{C_{e}},\\ \ln K_{c}=\left(\frac{\Delta S^{0}}{R}\right)-\left(\frac{\Delta H^{0}}{RT}\right), \end{array}$$

where Δ*G* ^0^, Δ*S* ^0^, and Δ*H* ^0^ are standard free energy change, standard enthalpy change, and standard entropy change, respectively; *K*_*c*_ is the equilibrium constant; *C*_*s*_ is the equilibrium concentration of AB25 on biosorbent (mg L^−1^); *C*_*e*_ is the equilibrium concentration of AB25 in solution (mg L^−1^); *R* is the ideal gas constant (8.314 J mol^−1^ K^−1^); and *T* is the biosorption temperature in Kelvin.

Langmuir isotherm model was applied to describe the biosorption of AB25. It is represented by the following equation;

(8) $$\begin{array}{c} \left(\frac{C_{e}}{q_{e}}\right)=\left(\frac{1}{Q_{\max}}K_{L}\right)+\left(\frac{C_{e}}{Q_{\max}}\right), \end{array}$$

where *Q*_max⁡_ is the maximum biosorption capacity of IJSP (mg g^−1^) and *K*_*L*_ is the Langmuir constant related to the biosorption energy (L mg^−1^).

*R*_*L*_, a dimensionless constant, was used to determine whether biosorption is favorable or not, which was calculated by

(9) $$\begin{array}{c} R_{L}=\frac{1}{1+K_{L}C_{0}}. \end{array}$$

Freundlich isotherm model was also applied to describe the biosorption of AB25. Linearized in logarithmic form of Freundlich isotherm model equation is represented by

(10) $$\begin{array}{c} \log q_{e}=\log K_{F}+\left(\frac{1}{n}\right)\log C_{0}, \end{array}$$

where *K*_*F*_ is the Freundlich constant and “1/*n*” is the heterogeneity factor.

## 4. Results and Discussion

### 4.1. Characterization of IJSP

#### 4.1.1. FTIR Analysis

**Figure 2:**
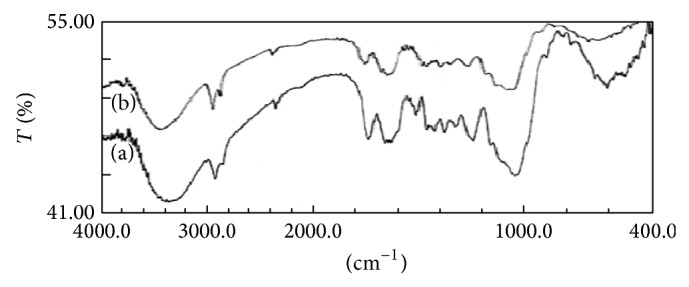
FTIR spectrum of IJSP alone and AB25 loaded IJSP.

The biosorption capacity of IJSP depends on physical as well as chemical reactivity of functional groups at surface. This reactivity creates an imbalance between forces at the surface when compared to those within the body, thus leading to molecular biosorption by the Van der Waals forces. Knowledge on surface functional groups would give insight to the biosorption capability of the IJSP. The spectra of IJSP and AB25 loaded IJSP were shown in Figure 2. The main peak at 3761 & 3416 cm^−1^ in the spectra of IJSP was due to the presence of bounded hydroxyl (–OH) or amine (–NH) groups. The peak at 2925 cm^−1^ is attributed to the symmetric and asymmetric C–H stretching vibration of aliphatic acids [40]. The peak observed at 1741 cm^−1^ is the stretching vibration of bond due to nonionic carboxyl groups (–COOH, –COOCH_3_) and may be assigned to carboxylic acids or their esters [40]. The bands at 1246 cm^−1^ indicate the C–O stretching in ether or alcohol and methoxy group, respectively. The bands 1649 cm^−1^ indicate functional group region of C=O, C–O, and O–H groups that exist as functional groups of IJSP. The peak at 1519 cm^−1^ is assigned to a conjugated hydrogen bonded carbonyl group. The peak at 1460 cm^−1^ indicates the presence of carboxyl groups (–COOH). The peaks at 1325 and 1378 cm^−1^ indicate the presence of C–H aliphatic bending. The peak at 1246 cm^−1^ indicates the presence of C–N from amine. The peaks at 1048 and 604 cm^−1^ indicate the presence of alkyl halide (C–F, C–Cl).

The spectra of IJSP and AB25 loaded IJSP have shown shifting of some peaks to higher frequency and decrease in intensity, lower than before biosorption, suggesting the involvement of functional groups (C=O, C–O, and O–H) in binding of AB25, as is shown in Table 1.

#### 4.1.2. SEM Micrographs

**Figure 3:**
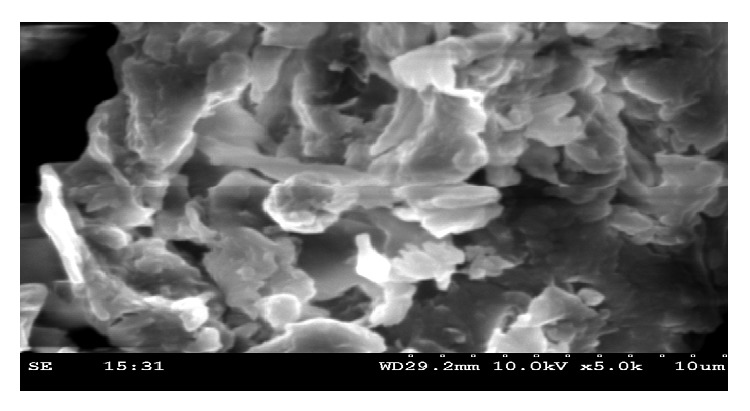
Scanning electron microscopic photograph of IJSP.

**Figure 4:**
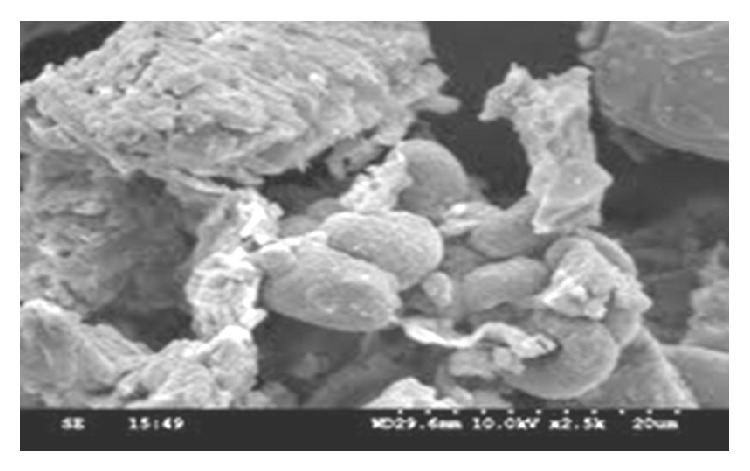
Scanning electron microscopic photograph of AB25 loaded IJSP.

Figures 3 and 4 show the SEM micrographs of IJSP sample before and after AB25 adsorption. It is clear that IJSP has considerable number of heterogeneous layers of pores. Thus, there is a good possibility for AB25 to be adsorbed. The surface of AB25 loaded IJSP, however, clearly shows that the surface of IJSP is covered with AB25 molecules. It is clearly observed in Figure 4.

#### 4.1.3. Point of Zero Charge (pH_PZC_)

**Figure 5:**
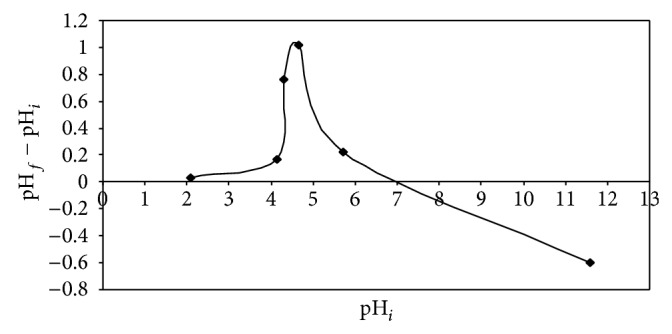
Determination of the point of zero charge of IJSP.

The point of zero charge for IJSP is found to be 7.  Figure 5 shows the plot between ΔpH, that is, (ΔpH = pH_*i*_ − pH_*f*_) and pH_*i*_. It is obvious from Figure 5 that the surface charge of the IJSP around pH 7 is zero. Hence, the pH_PZC_ at point of zero charge of the IJSP is 7.

### 4.2. Effect of pH

**Figure 6:**
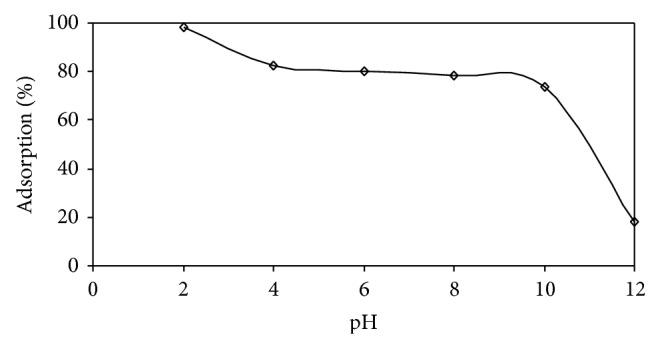
Effect of pH on equilibrium uptake of AB25. Conditions: agitation time = 3 h; *C*_0_ = 50 mg L^−1^; *V* = 0.025 L; Temp. = 35 ± 1°C; speed of agitation = 180 rpm; size of IJSP = <53 *μ*m; and dose = 100 mg.

The effect of pH is shown in Figure 6. The dye AB25 is acidic in nature. This dye (AB25) is also called anionic dye because it has −ve structure of the chromophore group.

One of the important parameters, affecting biosorption, is pH. Experiments have been carried out, with pH values ranging from 2 to 12 at temperature of 35°C, using 100 mg of biosorbent. It can be observed that the process of adsorption is highly dependent on the pH value of the solution. The removal efficiencies of AB25 dye decrease, with an increase in the value of the pH of the solution.

The highest removal efficiency was achieved at around pH 2 and thereafter the removal decreased. This may be due to the high electrostatic attraction between the positively charged surface of IJSP and AB25, which is anionic in nature. Besides the above, the presence of –SO_3_ group in dye (AB25) is largely responsible for increasing the dye adsorption process as the initial increase in pH may lead to an increase in the more −ve charged sites on the biosorbent surfaces and a decrease in the number of +ve charged sites. Due to electrostatic repulsion, the −ve charged surface is not in favor of biosorption of AB25 anions.

The effect of a decrease in the pH of the system is to cause a decrease in the number of −ve charged sites and an increase in the number of +ve charged sites. This, in fact, is one reason which favors the adsorption of dye anions because of the electrostatic attraction. The same behavior was observed in case of durian peel towards the acidic dye [41]. Similar studies were reported in the use of calcinated colemanite ore waste [42] and acid 183 and acid green 25 onto shell of bittim [43].

The uptake of AB25 was higher around value of 2 for pH, but there were no significant changes in the pH range 4–7. The high value of uptake of AB25 may be due to the excessive +ve change on dye anion AB25 for the biosorption sites.

With an increase in the pH values above that of PZC value (more than 7), the result was that the biosorbent surface became predominantly −ve charged which resulted in the enhancement of electrostatic repulsion between IJSP surface and dye anions.

Studies have reported a similar behavior by the adsorption of reactive orange 16 on nonactivated Brazilian pine fruit shell and adsorption of AB25 dye on biogas residual slurry [44].

### 4.3. Effect of IJSP Dose

**Figure 7:**
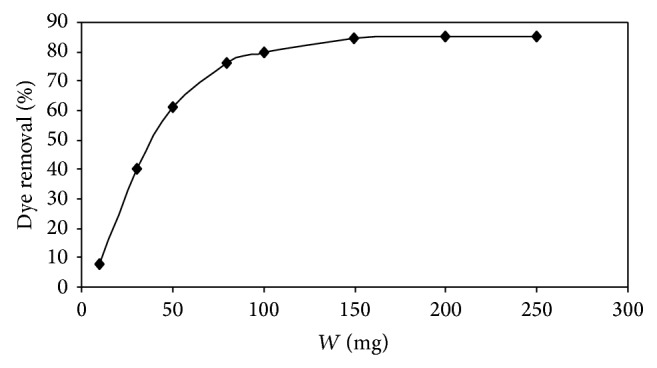
Effect of adsorbent dose on the adsorption of AB25 onto IJSP. Conditions as in Figure 6 except dose and pH = 5.44.

The effect of biosorbent (IJSP) dose on the removal of AB25 from the aqueous solution is shown in Figure 7. The figure reveals that the removal of AB25 increases up to a certain limit (100 mg), and then it remains almost constant. An increase in biosorption with biosorbent dose can be attributed to increased surface area and the availability of more adsorption sites [10]. But the amount adsorbed per unit mass of the biosorbent decreases considerably. The decrease in unit biosorption, with increasing dose of adsorbent, is basically due to the biosorption sites remaining, unsaturated during the biosorption process. For the quantitative removal of AB25 from 25 mL of 50 mg L^−1^ a maximum dose of 100 mg of biosorbent is required. It is shown in Figure 7.

### 4.4. Kinetic Study

#### 4.4.1. Effect of Initial AB25 Concentration and Contact Time

**Figure 8:**
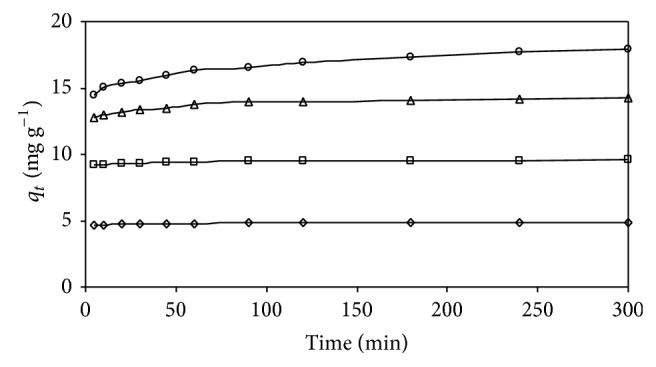
Effect of initial concentration and contact time on CR adsorption. Conditions: AB25 concentration: ◊: 25 mg L^−1^; □: 50 mg L^−1^; △: 75 mg L^−1^; ○: 100 mg L^−1^; and as in Figure 7.

**Figure 9:**
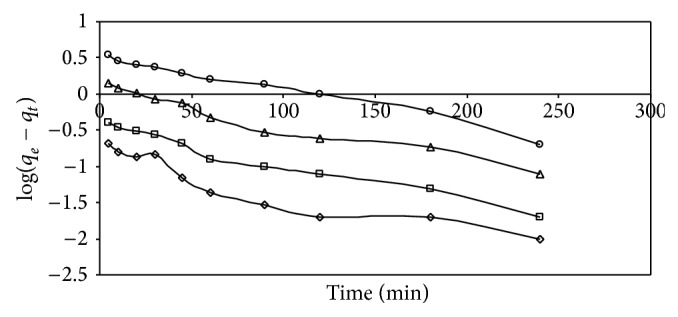
Pseudo-first-order model for AB25 on IJSP for different initial concentrations. Conditions as in Figure 8.

**Figure 10:**
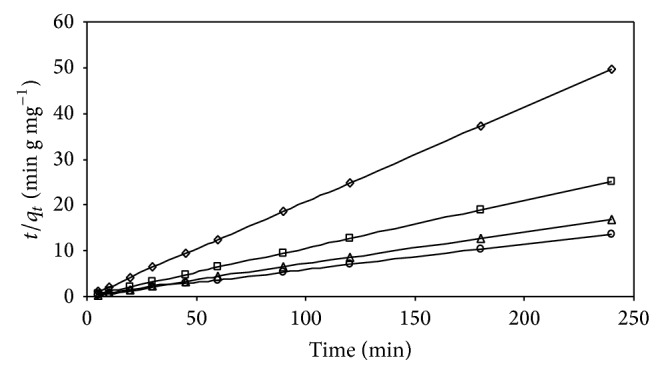
Pseudo-second-order model for AB25 on IJSP for different initial concentrations. Conditions as in Figure 8.

The biosorption of AB25 on IJSP was studied at different AB25 concentrations (range of 25–100 mg L^−1^).  Figure 8 shows the result for effect of initial concentration on biosorption of AB25 on IJSP at 35°C. It was observed that AB25 uptake was rapid, for the first 45 minutes, and thereafter it proceeded at a slower rate and finally attained saturation.  Figure 8 also indicates that an increase in initial AB25 concentration leads to an increase in the biosorption of AB25, on IJSP. The equilibrium biosorption increases from 4.84 to 17.71 mg g^−1^, with increase in the initial AB25 concentration from 25 to 100 mg L^−1^. As the initial AB25 concentration increased from 25 to 100 mg L^−1^, the equilibrium removal of AB25 registered a decrease from 77.41% to 70.86%. At lower concentrations, solute concentrations to biosorbent sites ratio is higher, due to an increase in AB25 removal. At higher concentrations, lower biosorption yield is due to the saturation of adsorption sites. The AB25 reached equilibrium approximately at 0.5, 0.75, 1.5, and 2 hours for AB25 concentrations of 25, 50, 75, and 100 mg L^−1^, respectively. However, the experimental data were obtained at 300 min to be sure that full equilibrium conditions were attained. Data on the adsorption kinetics of AB25 using various biosorbents has shown similar range of biosorption rates. Studies on the adsorption of AB25 on hazel nut shell [7], spent brewery grains [5], and diatomite [10] reported that the equilibrium was reached in about 3 h time.

(*1) Biosorption Kinetics*. For evaluating the biosorption kinetics of AB25, the pseudo-first-order and pseudo-second-order kinetic models were used, to fit the experimental data. Using (3) and (4), a log(*q*_*e*_ − *q*_*t*_) versus *t* plot was prepared for different AB25 concentrations (Figure 9). The pseudo-first-order model data does not fit as straight lines for most initial concentrations, indicating that this model is less appropriate. The Lagergren first-order rate constant (*k*_1_) was calculated from the model, and the values are presented in Table 2 along with the corresponding correlation coefficients. The experimental kinetic data were further analyzed, using the pseudo-second-order model by plotting *t*/*q*_*t*_ against *t* for different initial AB25 concentrations (Figure 10). A straight line was obtained in all the above cases. Using (4), the second-order rate constant (*k*_2_) and *q*_*e*_ values were determined from the plots. The values of correlation coefficient were found to be very high (*R* ^2^ > 0.9999) and the theoretical *q*_*e* cal_ values obtained from this model were closer to the experimental *q*_*e* exp⁡_ values at different initial AB25 concentrations (Table 2). It is important to note that, for the pseudo-first-order model, the correlation coefficient obtained in this study is *R* ^2^ < 0.9517 at different initial AB25 concentrations, which is lower, when compared with the correlation coefficient, obtained from the pseudo-second-order model. Moreover, from Table 2, it can be seen that the experimental values of *q*_*e* exp⁡_ are not in good agreement with the theoretical values calculated (*q*_*e* cal_) from the pseudo-first-order equation. Therefore, it can be concluded that the pseudo-second-order kinetic model provides a better correlation for the adsorption of AB25 on IJSP at different initial AB25 concentrations, compared to the pseudo-first-order model. Similar results were reported for the adsorption of AB25 and AY17 on spent brewery grains [5] and adsorption of AB25 and methylene blue on Hazelnut shells [7].

The pseudo-first-order and pseudo-second-order kinetic models could not identify the diffusion mechanism. Therefore, the kinetic results were analyzed by using the intraparticle diffusion model. Weber and Morris model was used through (5) to investigate the intraparticle diffusion mechanism, by plotting a graph between *q*_*t*_ and *t* ^0.5^ (the figure is not shown). If the intraparticle diffusion was the only rate-controlled step, the plot passed through the origin. If not, the boundary layer diffusion controlled the biosorption to some extent. The plots obtained were not linear over the whole time range, implying that more than one process affected the adsorption process. The data obtained are arranged in Table 3.

A similar behavior was reported for the removal of Congo red by using cattail root [4]. Table 3 shows at least three regions that represent boundary layer diffusion, followed by intraparticle diffusion in macro-, meso-, and micropores. These three regions are followed by a horizontal line representing the system at equilibrium.

From Table 3, it is observed that there are three linear regions. At the beginning of adsorption, there is a linear region which represents the rapid surface loading, followed by the second linear region which represents pore diffusion, and the third and final horizontal linear region which represents the equilibrium condition. The Microsoft Excel 2003 software package was used to analyze various regions available in the graph and results of linear regression are arranged in Table 3 for various initial concentrations. The intraparticle diffusion parameter, *k*_*pi*_, is determined from the slope of each region, while the intercept of each region is proportional to the boundary-layer thickness. The calculated values of *k*_*pi*_ and the intercept, *C*_*i*_, for all the linear regions are given in Table 3.

(*2) Effect of Temperature. *To study the effect of temperature on the influence of removal of AB25, using IJSP, several experiments were carried out at temperature values of 35, 45, 55, and 65°C; pH value of 5.44; and the AB25 concentration value of 50 mg L^−1^ with the adsorption dosage at 4 g L^−1^.

It was found that the efficiency of removal of AB25 decreases with the temperature from 35 to 65°C at the same dye concentration. The process is termed exothermic, wherein the increase in temperature results in a decrease in the value of *Q*_max⁡_.

Experimental results indicated that adsorption of AB25 gets decreased from high of 76.52 to 53.86% for the initial concentration of 50 mg L^−1^, when the temperature rises from 35 to 65°C. This may be due to the decrease in the surface activity which suggests that the process of adsorption between AB25 and IJSP is exothermic in nature.

To study the effect of temperature on the adsorption of AB25 on ISJP, two models, namely, pseudo-first-order and pseudo-second-order kinetic models, were used to fit the data obtained from conducting the experiments.

By using (3) and (4) equations, a log(*q*_*e*_ − *q*_*t*_) versus  *t* plot was prepared for various temperatures (the figure is not drawn). The plot indicates that this model is less appropriate for the relationship. The Lagergren first-order-rate constant, *k*, calculated from the model, is presented in Table 4, in which the corresponding correlation coefficients are also shown.

#### 4.4.2. Thermodynamic Study

Using (6) and (7), the various thermodynamic parameters, namely, (standard free energy change) Δ*G* ^0^, (standard enthalpy change) Δ*H* ^0^, and (standard entropy change) Δ*S* ^0^, and change of adsorption were evaluated. The adsorption process is essentially temperature dependent, and a study on this dependence provides valuable and useful information on one of the parameters, namely, enthalpy, during the adsorption.

**Figure 11:**
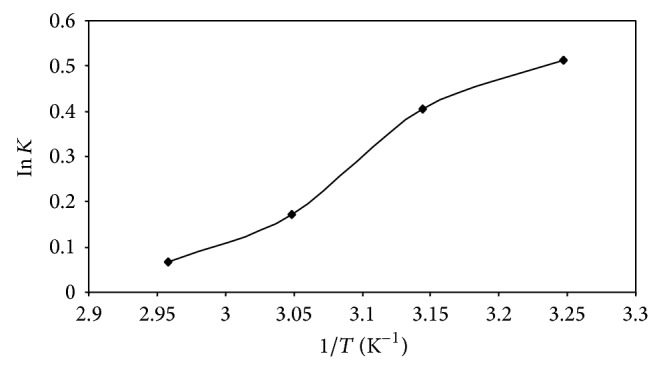
Plot of ln *K*_*c*_ against *T* ^−1^ for the removal of AB25 by IJSP. Conditions as in Figure 8.

A plot between ln *K*_*c*_ and *T* ^−1^ of the AB25 adsorption process was prepared, which is indicated in Figure 11. The slope and intercept values obtained by using a curve-fitting program were used to calculate the values of Δ*H* ^0^ and Δ*S* ^0^. The values of slope and intercept are given by Δ*H* ^0^/*R* and Δ*S* ^0^/*R*, respectively. The standard free energy change (Δ*G* ^0^) along with standard entropy change (Δ*H* ^0^) and entropy (Δ*H* ^0^) are shown in Table 5.

It was found that the equilibrium constant values decreased with an increase in the temperature. The overall value of Δ*G* ^0^ was found to be negative and it increased with an increase in temperature, as listed in Table 5. Negative values are indication of the spontaneous nature of all the adsorption processes and rapid and more spontaneous adsorption. The negative value of Δ*S* ^0^ is suggesting the randomness at the solid or solution interface during the process of adsorption.

The variation of Δ*G* ^0^ and its trend demonstrate that adsorption of AB25 by IJSP is more spontaneous and more favorable, especially at low temperatures in the range used in the study. This fact was confirmed by Δ*H* ^0^ being positive, which indicates the exothermic nature of the adsorption process [45].

#### 4.4.3. Equilibrium Modeling

**Figure 12:**
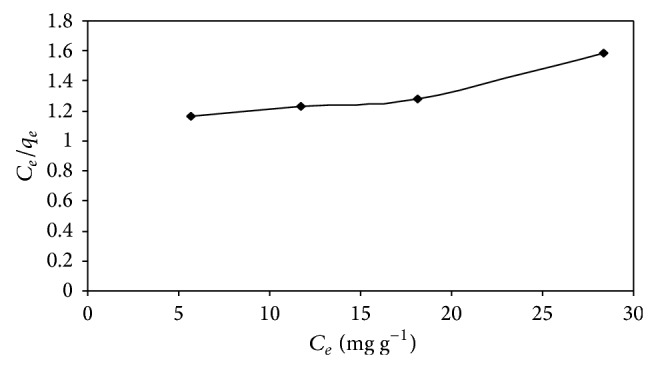
Langmuir isotherm of AB25 on IJSP at 35 ± 1°C. Conditions as in Figure 8.

**Figure 13:**
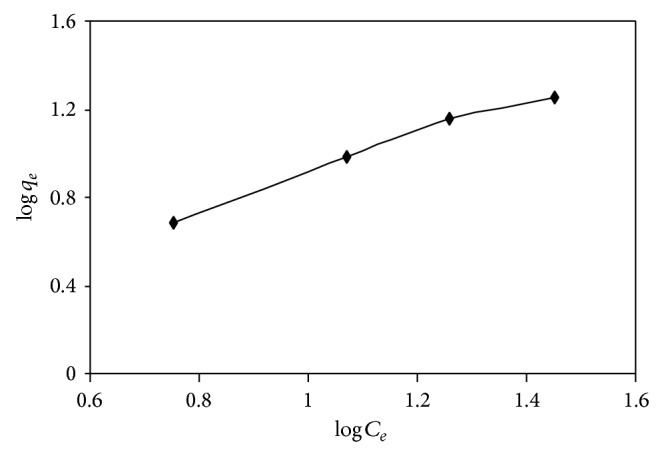
Freundlich isotherm of AB25 on IJSP at 35 ± 1°C. Conditions as in Figure 8.

Several mathematical models can be used, to describe the experimental data of adsorption isotherms. In this work, the equilibrium data at different concentrations of AB25 and the adsorption of AB25 on IJSP were modeled with the the Langmuir and Freundlich models. The details of the Langmuir and Freundlich isotherms are given in (8) and (10) and their respective plots are shown in Figures 12 and 13, respectively. The values of the Langmuir and Freundlich constants, obtained during these studies, are presented in Table 6. Both Langmuir and Freundlich models are well suited for the experimental data of AB25 on IJSP, as the coefficients of correlation indicate.

The essential characteristics of the Langmuir isotherm can be expressed in terms of a dimensionless constant, separation factor *R*_*L*_, that is given by (9) [4]. The value of *R*_*L*_ indicates the shape of the isotherm to be either unfavorable (*R*_*L*_ > 1), linear (*R*_*L*_ = 1), favorable (0 < *R*_*L*_ < 1), or irreversible (*R*_*L*_ = 0). The *R*_*L*_ value for the adsorption of AB25 on IJSP was found to be 0.0124, which indicates that the adsorption is a favorable process.

Similar to the above calculations, for various concentrations of AB25, both equilibrium models, the Langmuir model and the Freundlich model, were extended to understand the effect of temperature of AB25 solutions on IJSP. The values of the Langmuir and Freundlich constants, calculated from the figures (figures are not shown) similar to Figures 12 and 13, are presented in Table 6. Negative values for the Langmuir and Freundlich isotherm constants in Table 6 indicate the inadequacy of the isotherm model to explain the sorption process. These kinds of negative values are available in the literature [46–49].

## 5. Comparison of Various Low-Cost Adsorbents

The adsorption capacity of IJSP for the removal of AB25 is shown in Table 7. The adsorption capacity of various adsorbents for the removal of AB25 is also shown in Table 7. For comparison purpose, the removal capacity of IJSP compares well with that of the other adsorbents used. The adsorption capacity of IJSP for AB25 was found to be 54.95 mg g^−1^, which is higher than the adsorption capacity of various low-cost adsorbents for AB25, reported in the literature. It can be concluded that IJSP was extremely effective in the removal of AB25 from aqueous solution when compared with the effectiveness of other adsorbents.

## 6. Desorption

**Figure 14:**
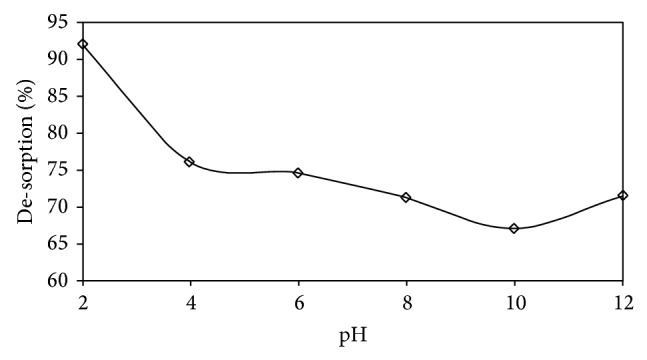
Desorption studies for the removal of AB25. Conditions as in Figure 6.

The mechanism of the process of adsorption and the recovery of both adsorbate and adsorbent are better elucidated by making use of the desorption studies. In many cases, the treatment process of desorption is just opposite to the adsorption process in the pH effect. The above observation is illustrated in Figure 14. The percent desorption was found to decrease from 92.06 to 71.52 with an increase in the pH value from 2 to 12. The decrease in percent desorption indicates that the adsorption of AB25 is primarily due to ion exchange and physical adsorption process [24]. Studies have revealed that, in case of desorption of rhodamine-B from the surface of coir pith [50], a similar pattern is observed.

## 7. Conclusions

The present study has revealed that IJSP is a low-cost and eco-friendly biosorbent for the removal of AB25 from aqueous solution.

The study has revealed that the dye adsorbed varies with the initial concentration of AB25 and contact time. The investigations aimed at finding the effect of operation variables reveal the Langmuir adsorption capacity. Pseudo-second-order kinetics was used to predict the biosorption kinetics as it is the best for the biosorption of AB25 by IJSP, with good correlation.

Results of present study have been satisfactory and they indicate the potential of IJSP for removal of AB25 from the aqueous solution.

## Figures and Tables

**Table 1:** FTIR of IJSP adsorbent alone and AB25 loaded IJSP.

IR peak	Frequency (cm^−1^)	
	Before adsorption	After adsorption
1	3761.36, 3416.19	3433.60
2	2925.69	2920.49, 2856.83
3	1741.21	1743.81
4	1649.93	1624.21, 1601.06
5	1519.51	1512.33
6	1460.03	1462.17
7	1378.50	1377.30
8	1325.97	1323.29
9	1246.28	1249.99
10	1048.74	1165.11

**Table 2:** Pseudo-first-order and pseudo-second-order rate constants at 35°C and different initial AB25 concentrations. Conditions: size of IJSP = <53 *μ*m; pH of AB25 soln = 5.44; dose of IJSP = 100 mg; and speed of agitation = 180 rpm.

*C*_0_ (mg L^−1^)	*q*_*e* exp⁡_ (mg g^−1^)	Pseudo-first-order model			Pseudo-second-order model		
		*k*_1_ (min^−1^)	*q*_*e* cal_ (mg g^−1^)	*R* ^2^	*K*_2_ (min^−1^)	*q*_*e* cal_ (mg g^−1^)	*R* ^2^
25	4.8477	0.0237	5.4967	0.9517	0.3919	4.8498	1.0000
50	9.5645	0.0122	10.7997	0.9726	0.1574	9.5698	1.0000
75	14.2129	0.0182	15.4760	0.9890	0.0461	14.2309	0.9999
100	17.9102	0.0092	21.0927	0.9924	0.0146	17.9673	0.9993

**Table 3:** Intraparticle diffusion constants for different initial AB25 concentrations at 30°C. Conditions as in Table 2.

Linear portion ↓	Constants ↓	*C*_*o*_ = 25_ _mg/L	*C*_*o*_ = 50_ _mg/L	*C*_*o*_ = 75_ _mg/L	*C*_*o*_ = 100_ _mg/L
First	*K*_*P*1_ (mg g^−1^ min^−0.5^)	0.0272	0.0421	0.1537	0.3818
	*C*_1_ (mg g^−1^)	4.5918	9.0716	12.4920	13.7240
	*R* ^2^	0.9799	0.9893	0.9904	0.9189
Second	*K*_*P*2_ (mg g^−1^ min^−0.5^)	0.0094	0.0131	0.0372	0.3009
	*C*_2_ (mg g^−1^)	4.7270	9.3399	13.5570	13.9750
	*R* ^2^	0.9777	0.9966	0.9812	0.9843
Third	*K*_*P*3_ (mg g^−1^ min^−0.5^)	0.0050	—	—	0.1712
	*C*_3_ (mg g^−1^)	4.7602	—	—	15.0050
	*R* ^2^	0.9989	—	—	0.9910

**Table 4:** Pseudo-first-order and pseudo-second-order rate constants for different temperatures. Conditions as in Table 2 except temperature.

Temperature (°C)	*q*_*e* exp⁡_ (mg g^−1^)	Pseudo-first-order model			Pseudo-second-order model		
		*k*_1_ (min^−1^)	*q*_*e* cal_ (mg g^−1^)	*R* ^2^	*K* ^2^ (min^−1^)	*q*_*e* cal_ (mg g^−1^)	*R* ^2^
35	9.5645	0.0138	9.7181	0.9517	0.1514	9.5602	1.0000
45	8.9688	0.0069	10.2594	0.9726	−0.1144	9.1912	0.9999
55	7.4648	0.0092	7.9676	0.989	−0.0417	7.9745	0.9999
65	6.7324	0.0046	8.9897	0.9924	−0.0375	7.1480	0.9993

**Table 5:** Thermodynamic parameters for the adsorption of AB25 on IJSP Conditions as in Table 4.

Temperature (°C)	*K*_*c*_ = *C*_*s*_/*C*_*e*_	Δ*G* ° (kJ mole^−1^)	Δ*S* ° (kJ mole^−1^ K^−1^)	Δ*H* ° (kJ mole^−1^)
35	3.2581	−3.0253	−0.0915	−31.2846
45	2.5398	−2.4647		
55	1.4825	−1.0739		
65	1.1673	−0.4349		

**Table 6:** Biosorption isothermal parameter values for the removal of AB25 on IJSP at different concentrations of AB25 and temperatures of AB25 solution. Conditions as in Tables 2 and 4.

Parameters	Langmuir constants			Freundlich constants		
	*Q*_max⁡_ (mg g^−1^)	*K*_*L*_ (L mg^−1^)	*R* ^2^	*K*_*F*_	*n*	*R* ^2^
Conc. of AB25 (mg L^−1^)	54.9451	0.0178	0.9065	1.2030	1.2085	0.9875
Temperature (°C)	5.1813	−0.1755	0.9936	1.2030	−1.9361	0.9870

**Table 7:** Adsorption capacities of AB25 on various adsorbents.

S. number	Name of adsorbent	*q*_*e*_ (mg g^−1^)	Reference
1	IJSP	54.95	Present study
2	Hazelnut	40.80	[7]
3	Walnut	36.98	[7]
4	Cherry	31.98	[7]
5	Oak	27.85	[7]
6	Pitch-pine	26.19	[7]
7	Spent brewery grains	24.02	[5]
8	Egyptian bagasse pith	17.50	[8]
9	Wood sawdust	5.99	[9]
10	Diatomite	21.41	[10]
11	Peat	12. 7	[11]
12	Wood	11.60	[12]
13	Maize cob	41.40	[12]
14	Bagasse pith	21.70	[12]
15	Char from bamboo	16.91	[13]
16	Peat	8.9–16.3	[14]
17	Modified silica	45.8	[15]

## References

[1] Moussavi G., Khosravi R. (2011). The removal of cationic dyes from aqueous solutions by adsorption onto pistachio hull waste. *Chemical Engineering Research and Design*.

[2] Anbia M., Hariri S. A. (2010). Removal of methylene blue from aqueous solution using nanoporous SBA-3. *Desalination*.

[3] Hameed B. H., Hakimi H. (2008). Utilization of durian (*Durio zibethinus* Murray) peel as low cost sorbent for the removal of acid dye from aqueous solutions. *Biochemical Engineering Journal*.

[4] Hu Z., Chen H., Ji F., Yuan S. (2010). Removal of Congo Red from aqueous solution by cattail root. *Journal of Hazardous Materials*.

[5] Jaikumar V., Sathish Kumar K., Gnana Praksh D. (2009). Biosorption of acid dyes using spent brewery grains: characterization and modeling. *International Journal of Applied Science and Engineering*.

[6] dos Santos A. B., Cervantes F. J., van Lier J. B. (2007). Review paper on current technologies for decolourisation of textile wastewaters: perspectives for anaerobic biotechnology. *Bioresource Technology*.

[7] Ferrero F. (2007). Dye removal by low cost adsorbents: Hazelnut shells in comparison with wood sawdust. *Journal of Hazardous Materials*.

[8] Chen B., Hui C. W., McKay G. (2001). Film-pore diffusion modeling and contact time optimization for the adsorption of dyestuffs on pith. *Chemical Engineering Journal*.

[9] Ho Y. S., McKay G. (1998). Sorption of dye from aqueous solution by peat. *Chemical Engineering Journal*.

[10] Badii K., Farama D. A., Saberi M. A., Limaee N. Y., Shafaei S. Z. (2010). Adsorption of Acid blue 25 dye on diatomite in aqueous solutions. *Indian Journal of Chemical Technology*.

[11] Ho Y. S., McKay G. (1998). A kinetic models for the sorption of dye from aqueous solution by wood. *Transactions of IChemE B*.

[12] Gupta V. K. (2009). Application of low-cost adsorbents for dye removal—a review. *Journal of Environmental Management*.

[13] Mui E. L. K., Cheung W. H., Valix M., McKay G. (2010). Dye adsorption onto char from bamboo. *Journal of Hazardous Materials*.

[14] Poots V. J. P., McKay G., Healy J. J. (1976). The removal of acid dye from effluent using natural adsorbents. I. Peat. *Water Research*.

[15] Phan T. N. T., Bacquet M., Morcellet M. (2000). Synthesis and characterization of silica gels functionalized with monochlorotriazinyl *β*-cyclodextrin and their sorption capacities towards organic compounds. *Journal of Inclusion Phenomena*.

[16] Panda G. C., Das S. K., Guha A. K. (2009). Jute stick powder as a potential biomass for the removal of congo red and rhodamine B from their aqueous solution. *Journal of Hazardous Materials*.

[17] Doǧan M., Abak H., Alkan M. (2008). Biosorption of methylene blue from aqueous solutions by hazelnut shells: equilibrium, parameters and isotherms. *Water, Air, and Soil Pollution*.

[18] Patil A. K., Shrivastava V. S. (2010). Alternanthera bettzichiana plant powder as low cost adsorbent for removal of Congo red from aqueous solution. *International Journal of ChemTech Research*.

[19] Gong R. M., Ding Y., Li M., Yang C., Liu H. J., Sun Y. Z. (2005). Utilization of powdered peanut hull as biosorbent for removal of anionic dyes from aqueous solution. *Dyes and Pigments*.

[20] Ponnusami V., Vikram S., Srivastava S. N. (2008). Guava (Psidium guajava) leaf powder: novel adsorbent for removal of methylene blue from aqueous solutions. *Journal of Hazardous Materials*.

[21] Sivaramakrishna L., Sivasankar Reddy M., Jagadeesh M., Wan Zuhairi W. Y., Taha M. R., Varada Reddy A. (2014). Evaluation of biomass, Indian Jujuba Seed (IJS) for removal of Congo red. *American Journal of Environmental Sciences*.

[22] Hameed B. H. (2009). Removal of cationic dye from aqueous solution using jackfruit peel as non-conventional low-cost adsorbent. *Journal of Hazardous Materials*.

[23] Arami M., Limaee N. Y., Mahmoodi N. M., Tabrizi N. S. (2006). Equilibrium and kinetics studies for the adsorption of direct and acid dyes from aqueous solution by soy meal hull. *Journal of Hazardous Materials B*.

[24] Namasivayam C., Muniasamy N., Gayatri K., Rani M., Ranganathan K. (1996). Removal of dyes from aqueous solutions by cellulosic waste orange peel. *Bioresource Technology*.

[25] Noreen S., Bhatti H. N., Nausheen S., Sadaf S., Ashfaq M. (2014). Batch and fixed bed adsorption study for the removal of Drimarine Black CL-B dye from aqueous solution using a lignocellulosic waste: a cost affective adsorbent. *Industrial Crops and Products*.

[26] Dawood S., Sen T. K. (2012). Removal of anionic dye Congo red from aqueous solution by raw pine and acid treated pine cone powder as adsorbent: equilibrium, thermodynamic, kinetics, mechanism and process design. *Water Research*.

[27] Benyoucef S., Amrani M. (2011). Adsorption of phosphate ions onto low cost Aleppo pine adsorbent. *Desalination*.

[28] Ponnusami V., Gunasekar V., Srivastava S. N. (2009). Kinetics of methylene blue removal from aqueous solution using gulmohar (Delonix regia) plant leaf powder: multivariate regression analysis. *Journal of Hazardous Materials*.

[29] Malekbala M. R., Hosseini S., Kazemi Yazdi S., Masoudi Soltani S., Malekbala M. R. (2012). The study of the potential capability of sugar beet pulp on the removal efficiency of two cationic dyes. *Chemical Engineering Research and Design*.

[30] Sen T. K., Afroze S., Ang H. M. (2011). Equilibrium, kinetics and mechanism of removal of methylene blue from aqueous solution by adsorption onto pine cone biomass of *Pinus radiata*. *Water, Air, & Soil Pollution*.

[31] Mahmoodi N. M., Hayati B., Arami M., Lan C. (2011). Adsorption of textile dyes on pine cone from colored wastewater: kinetic, equilibrium and thermodynamic studies. *Desalination*.

[32] Senthil Kumar P., Ramalingam S., Senthamarai C., Niranjanaa M., Vijayalakshmi P., Sivanesan S. (2010). Adsorption of dye from aqueous solution by cashew nut shell: Studies on equilibrium isotherm, kinetics and thermodynamics of interactions. *Desalination*.

[33] Abd EI-Latif M. M., Ibrahim A. M., EI-Kady M. F. (2010). Adsorption equilibrium, kinetics and thermodynamics of methylene blue from aqueous solutions using biopolymer oak sawdust composite. *The Journal of American Science*.

[34] Aksakal O., Ucun H. (2010). Equilibrium, kinetic and thermodynamic studies of the biosorption of textile dye (Reactive Red 195) onto *Pinus sylvestris* L.. *Journal of Hazardous Materials*.

[35] Tunali Akar S., Gorgulu A., Akar T., Celik S. (2011). Decolorization of Reactive Blue 49 contaminated solutions by Capsicum annuum seeds: Batch and continuous mode biosorption applications. *Chemical Engineering Journal*.

[36] Deniz F., Saygideger S. D. (2011). Removal of a hazardous azo dye (Basic Red 46) from aqueous solution by princess tree leaf. *Desalination*.

[37] Gulnaz O., Sahmurova A., Kama S. (2011). Removal of Reactive Red 198 from aqueous solution by *Potamogeton crispus*. *Chemical Engineering Journal*.

[38] Song J., Zou W., Bian Y., Su F., Han R. (2011). Adsorption characteristics of methylene blue by peanut husk in batch and column modes. *Desalination*.

[39] Somasekhara Reddy M. C., Sivaramakrishna L., Varada Reddy A. (2012). The use of an agricultural waste material, Jujuba seeds for the removal of anionic dye (Congo red) from aqueous medium. *Journal of Hazardous Materials*.

[40] Li F. T., Yang H., Zhao Y., Xu R. (2007). Novel modification pectin for heavy metal adsorption. *Chinese Chemical Letters*.

[41] Hameed B. H., Hakimi H. (2008). Utilization of durian (Durio zibethinus murray) peel as low cost sorbent for the removal of acid dye from aqueous solutions. *Biochemical Engineering Journal*.

[42] Atar N., Olgun A. (2007). Removal of acid blue 62 on aqueous solution using calcinated colemanite ore waste. *Journal of Hazardous Materials*.

[43] Aydin H., Baysal G. (2006). Adsorption of acid dyes in aqueous solutions by shells of bittim (Pistacia khinjuk Stocks). *Desalination*.

[44] Namasivayam C., Yamuna R. T. (1995). Adsorption of direct red 12 B by biogas residual slurry: equilibrium and rate processes. *Environmental Pollution*.

[45] Calvete T., Lima E. C., Cardoso N. F., Dias S. L. P., Pavan F. A. (2009). Application of carbon adsorbents prepared from the Brazilian pine-fruit-shell for the removal of Procion Red MX 3B from aqueous solution—Kinetic, equilibrium, and thermodynamic studies. *Chemical Engineering Journal*.

[46] Robinson T., Chandran B., Nigam P. (2002). Effect of pretreatments of three waste residues, wheat straw, corncobs and barley husks on dye adsorption. *Bioresource Technology*.

[47] Abdullah M. A., Chiang L., Nadeem M. (2009). Comparative evaluation of adsorption kinetics and isotherms of a natural product removal by Amberlite polymeric adsorbents. *Chemical Engineering Journal*.

[48] Ennil Köse T. (2008). Agricultural residue anion exchanger for removal of dyestuff from wastewater using full factorial design. *Desalination*.

[49] Hussain A., Ghafoor A., Anwar-Ul-Haq M., Nawaz M. (2003). Application of the Langmuir and Freundlich equations for P adsorption phenomenon in saline-sodic soils. *International Journal of Agriculture and Biology*.

[50] Namasivayam C., Radhika R., Suba S. (2001). Uptake of dyes by a promising locally available agricultural solid waste: coir pith. *Waste Management*.

